# Supplementary material for: Scale-free and oscillatory spectral measures of sleep stages in humans
Source: Front Neuroinform. 2022 Oct 3;16:989262. doi: 10.3389/fninf.2022.989262 (PMC9574340; doi:10.3389/fninf.2022.989262)
Supplement: Supplementary file 1 [file Data_Sheet_1.pdf]

# 1 SUPPLEMENTARY MATERIALS

## 1.1 EEG recording specifications

| Nap/ night | Subsample | Original setting/aim | Nr. of subjects (females) | Age range (ys) | Available EEG derivations (10-20 system) | Recording apparatus | Precision (bit) | Hardware prefiltering (Hz) | Sampling rate (Hz/channel) | Recording software |
|---|---|---|---|---|---|---|---|---|---|---|
| Night sleep | MPIP[1] – I | Lab/sleep & IQ | 95 (43) | 18–69 | Fp1, Fp2, Fpz, AF1, AF2, F3, F4, Fz, F7, F8, C3, C4, Cz, P3, P4, Pz, T3, T4, T5, T6, O1, O2 | Comlab 32 Digiltal SleepLab | 8 | 0.53–70 | 250 | Brainlab 3.3 |
| | MPIP[1] – II | | 20 (12) | | Fp1, Fp2, F3, F4, C3, C4, P3, P4, O1, O2 | | | | | |
| | PPCU[2] – I | Home/Williams syndrome study (controls included here) | 20 (14) | 6–28 | Fp1, Fp2, Fpz, F3, F4, F7, F8, Fz, C3, C4, Cz, T3, T4, T5, T6, P3, P4, Pz, O1, O2, Oz | SD-LTM 32BS (Micromed Ltd, Italy) | 22 | 0.15-250 (plus <463.3 Hz digital antialising filtering before downsampling from 4096 to 1024 Hz) | 1024 | BRAIN QUICK System PLUS (Micromed) |
| | PPCU[2] – II | Home/Adolescent sleep | 23 (12) | 15–22 | | | | | | |
| | SU[3] – I | Lab/sleep & IQ, sleep spindle methodology, wake-sleep transition analysis | 49 (19) | 17–55 | Fp1, Fp2, F3, F4, F7, F8, Fz, C3, C4, Cz, T3, T4, T5, T6, P3, P4, O1, O2 | Flat Style SLEEP La Mont Headbox, HBX32-SLP preamplifier (La Mont Medical) | 12 | 0.5–70 | 249 | Datalab (Medcare) |
| | SU[3] – II | Lab/nightmare study (controls included here) | 16 (7) | 19–21 | Fp1, Fp2, F3, F4, Fz, F7, F8, C3, C4, Cz, P3, P4, Pz, T3, T4, T5, T6, O1, O2 | Brain-Quick BQ132S (Micromed) | 12 | 0.33–1500 (plus <450 Hz anti-aliasing digital filtering before downsampling from 4096 to 1024 Hz) | 1024 | System 98 (Micromed) |
| | SU[3] – III | Lab/home/ children's dreaming | 29 (15) | 3.84–8.42 | | Brain-Quick BQ132S/ SD LTM 32BS (Micromed) | 12/22 | 0.33–1500/0.15-250 (plus <450/<463.3 Hz anti-aliasing digital filtering before downsampling from 4096 to 1024 Hz) | | System 98/System Plus Evolution (Micromed) |

*[1]Max Planck Inst tute of Psychiatry, Munich, Germany; [2]Pázmány Péter Catholic University, Budapest Hungary; [3]Semmelweis University Budapest, Hungary*

**Table 1.** Detailed information regarding the EEG recording institution, setting, hardware and software.

## 1.2 Alternative intercept rationale and definition

One of the biggest advantages of adopting a parametric model for describing EEG power spectra is that we can capture the spectral phenomena using significantly fewer variables compared to the original spectral data (2 aperiodic parameters + 3 per peak, whereas the minimal number of bins in the power spectra is generally around 256), however we found that the slope and the intercept parameters provided by the FOOOF method are correlated with an average correlation of: $< r >= 0.47$ (Pearson correlations were calculated between the two variables for all the corresponding sleep stages and EEG channels, then averaged in the Fisher z-space and inverse transformed). The alternative intercept was defined as the value of the power-law component at the frequency of the peak with the highest power, as suggested in an earlier study, in order to achieve the least correlation with the slope. The average correlation between slope and the alternative intercept was $< r >= -0.03$.

## 1.3 Between-stage correlations in the spectral slope

The strongly subject-specific nature of the spectral slope had been demonstrated before, however only in the wake, resting state. In order to test this specificity in the domain of sleep, we compared spectral slopes values between sleep stages and found that in general there is a positive correlation between all stages within individuals, furthermore that the correlation is stronger between subsequent sleep stages, see Figure 1. Correlations coefficients between non-identical stages were in the range of $0.2 < r < 0.8$, with overall average correlation of $< r >= 0.49$, while p-values were between $10^{-58}$ and $10^{-2}$.

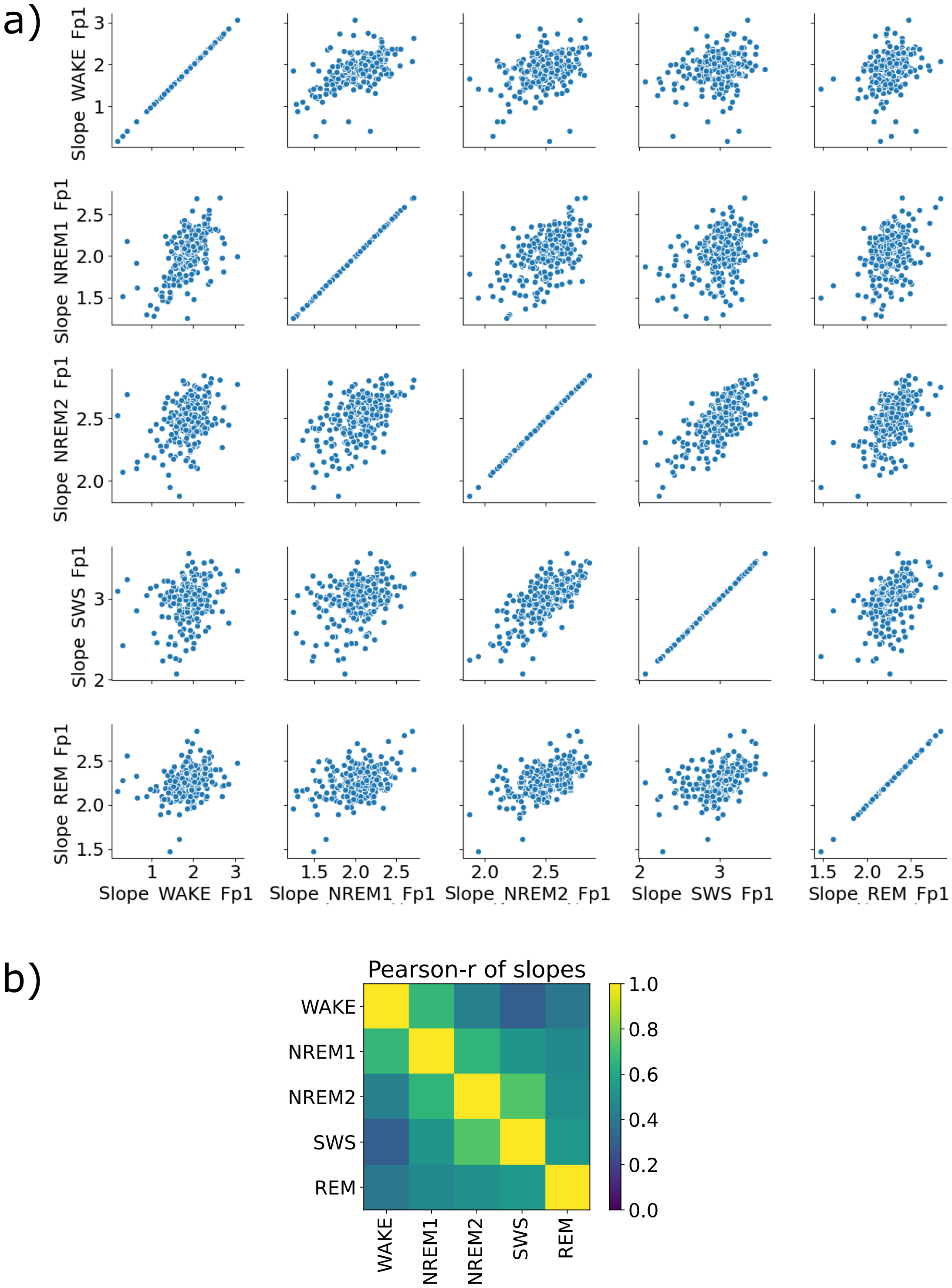


**Figure 1.** a) Covariance plot of spectral slopes between different sleep stages, each point corresponding to a subject. b) Pearson correlations of spectral slopes between sleep stages in the case of the Fp1 channel. It can be noted that the values closer to the main diagonal are higher, suggesting that there is more correlation between subsequent sleep stages.

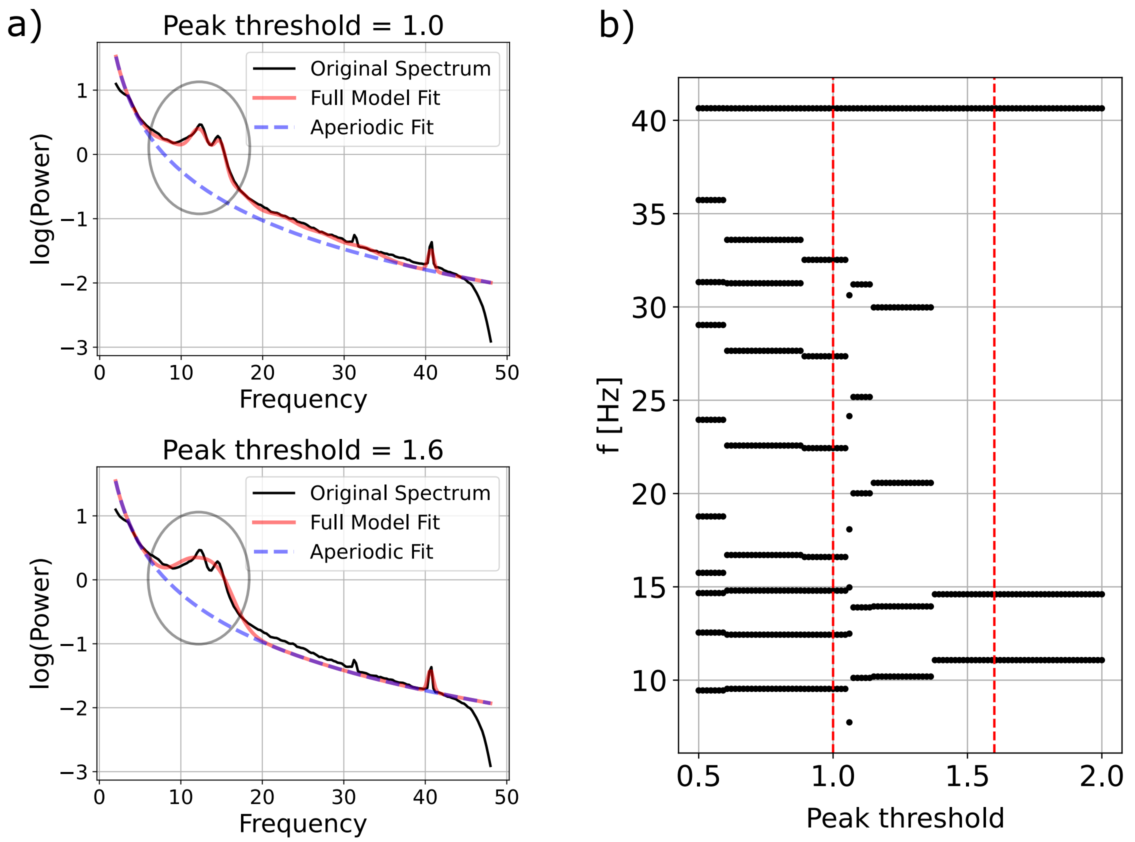


**Figure 2.** a) Two examples of fitting the same power spectrum using the FOOOF method with different peak threshold parameters, the model detects the double peak only for lower peak thresholds. b) Dependence of fitted peak frequencies on the peak threshold parameter. The vertical dashed lines represent the two cases on the left.

## 1.4 Challenges using the FOOOF method

Applying the FOOOF method to spectral data is straightforward, however by looking more closely at certain cases some undesired effects were noticed regarding the periodic component. In some instances when power spectra contained ‘double’ peaks, the model fitted them wrongly as a single wide peak (see below on Figure 2 a) subfigure’s lower row). In order to eliminate this issue the peak threshold parameter was decreased, which ultimately resulted in an acceptable fit, yet it was also an indication that more careful choice of control parameters might be needed than primarily expected. (The peak threshold determines the minimal deviation in power from the aperiodic component that is necessary for the data point to be considered as a peak candidate.) In order to investigate the effect of the peak threshold the same power spectrum had been fitted multiple times while varying the peak threshold quasi-continuously. On Figure 2 b) the frequencies of the found peaks were plotted in function of the peak threshold parameter, it had been expected that the number of found peaks increases as the threshold value descreases (as smaller irregularities in the power spectrum have a higher chance to be above this threshold), however a frequncy shift was also discovered in function of the peak threshold parameter. Knowing that the central peak frequencies are sensitive to this parameter choice a supervision of the fitting is advised.

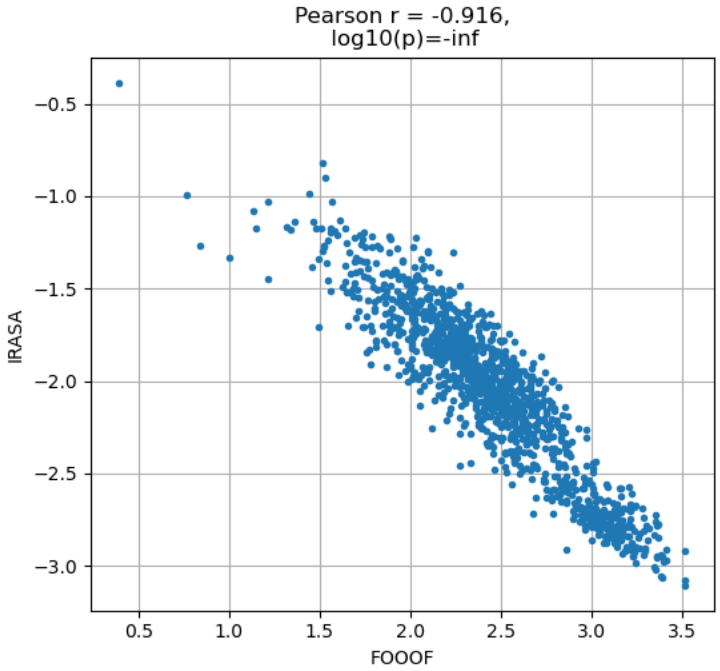


**Figure 3.** Pearson correlation of slope values calculated using the FOOOF and IRASA method.

### 1.5 Comparison of FOOOF slope values with IRASA

Another method that separates the aperiodic component of the spectrum is the irregular-resampling auto-spectral analysis (IRASA) [Wen and Liu, 2015]. As a partial validation of our results, we used an already available implementation of the IRASA method [Vallat and Walker, 2021] to calculate spectral slopes for the C3 EEG channel. A strong correlation was found between the slopes calculated with the two methods, see Figure 3.

## 1.6 Statistical results

### 1.6.1 Spectral slope

| Effect | Repeated Measures Analysis of Variance with Effect Sizes<br>Sigma-restricted parameterization<br>Effective hypothesis decomposition | | | | | |
|---|---|---|---|---|---|---|
| | SS | df | MS | F | p | $\eta_p^2$ |
| Intercept | 37765.20 | 1 | 37765.20 | 30064.52 | 0.00E+00 | 0.993 |
| {1}sex | 0.18 | 1 | 0.18 | 0.15 | 7.02E-01 | 0.001 |
| {2}age_group | 24.38 | 3 | 8.13 | 6.47 | 3.32E-04 | 0.086 |
| sex*age_group | 3.00 | 3 | 1.00 | 0.80 | 4.97E-01 | 0.011 |
| Error | 258.76 | 206 | 1.26 | | | |
| {3}STAGE | 882.03 | 4 | 220.51 | 770.29 | 0.00E+00 | 0.789 |
| STAGE*sex | 1.27 | 4 | 0.32 | 1.11 | 3.49E-01 | 0.005 |
| STAGE*age_group | 23.71 | 12 | 1.98 | 6.90 | 5.37E-12 | 0.091 |
| STAGE*sex*age_group | 3.79 | 12 | 0.32 | 1.10 | 3.54E-01 | 0.016 |
| Error | 235.88 | 824 | 0.29 | | | |
| {4}REGION | 22.42 | 4 | 5.60 | 113.33 | 0.00E+00 | 0.355 |
| REGION*sex | 0.36 | 4 | 0.09 | 1.83 | 1.21E-01 | 0.009 |
| REGION*age_group | 3.86 | 12 | 0.32 | 6.51 | 3.50E-11 | 0.087 |
| REGION*sex*age_group | 1.05 | 12 | 0.09 | 1.76 | 4.98E-02 | 0.025 |
| Error | 40.75 | 824 | 0.05 | | | |
| {5}LEFT-RIG | 0.08 | 1 | 0.08 | 2.56 | 1.11E-01 | 0.012 |
| LEFT-RIG*sex | 0.07 | 1 | 0.07 | 2.13 | 1.46E-01 | 0.010 |
| LEFT-RIG*age_group | 0.17 | 3 | 0.06 | 1.82 | 1.45E-01 | 0.026 |
| LEFT-RIG*sex*age_group | 0.27 | 3 | 0.09 | 2.95 | 3.39E-02 | 0.041 |
| Error | 6.32 | 206 | 0.03 | | | |
| STAGE*REGION | 5.54 | 16 | 0.35 | 55.23 | 0.00E+00 | 0.211 |
| STAGE*REGION*sex | 0.13 | 16 | 0.01 | 1.27 | 2.10E-01 | 0.006 |
| STAGE*REGION*age_group | 1.49 | 48 | 0.03 | 4.95 | 0.00E+00 | 0.067 |
| STAGE*REGION*sex*age_group | 0.47 | 48 | 0.01 | 1.55 | 9.54E-03 | 0.022 |
| Error | 20.68 | 3296 | 0.01 | | | |
| STAGE*LEFT-RIG | 0.05 | 4 | 0.01 | 1.52 | 1.93E-01 | 0.007 |
| STAGE*LEFT-RIG*sex | 0.11 | 4 | 0.03 | 3.42 | 8.82E-03 | 0.016 |
| STAGE*LEFT-RIG*age_group | 0.18 | 12 | 0.02 | 1.87 | 3.41E-02 | 0.027 |
| 3*5*1*2 | 0.30 | 12 | 0.02 | 3.09 | 2.75E-04 | 0.043 |
| Error | 6.66 | 824 | 0.01 | | | |
| REGION*LEFT-RIG | 0.49 | 4 | 0.12 | 10.09 | 5.60E-08 | 0.047 |
| REGION*LEFT-RIG*sex | 0.20 | 4 | 0.05 | 4.07 | 2.82E-03 | 0.019 |
| REGION*LEFT-RIG*age_group | 0.40 | 12 | 0.03 | 2.75 | 1.13E-03 | 0.039 |
| 4*5*1*2 | 0.38 | 12 | 0.03 | 2.65 | 1.75E-03 | 0.037 |
| Error | 9.95 | 824 | 0.01 | | | |
| STAGE*REGION*LEFT-RIG | 0.03 | 16 | 0.00 | 1.27 | 2.04E-01 | 0.006 |
| STAGE*REGION*LEFT-RIG*sex | 0.04 | 16 | 0.00 | 1.70 | 3.96E-02 | 0.008 |
| 3*4*5*2 | 0.08 | 48 | 0.00 | 0.98 | 5.08E-01 | 0.014 |
| 3*4*5*1*2 | 0.17 | 48 | 0.00 | 2.11 | 1.48E-05 | 0.030 |
| Error | 5.42 | 3296 | 0.00 | | | |

### 1.6.2 Intercept

| Effect | Repeated Measures Analysis of Variance with Effect Sizes<br>Sigma-restricted parameterization<br>Effective hypothesis decomposition | | | | | |
|---|---|---|---|---|---|---|
| | SS | df | MS | F | p | $\eta_p^2$ |
| Intercept | 318113.41 | 1 | 318113.41 | 23895.92 | 0.00E+00 | 0.991 |
| {1}sex | 19.18 | 1 | 19.18 | 1.44 | 2.31E-01 | 0.007 |
| {2}age_group | 1053.33 | 3 | 351.11 | 26.37 | 1.65E-14 | 0.275 |
| sex*age_group | 20.79 | 3 | 6.93 | 0.52 | 6.69E-01 | 0.007 |
| Error | 2782.30 | 209 | 13.31 | | | |
| {3}STAGE | 96.09 | 4 | 24.02 | 35.73 | 0.00E+00 | 0.146 |
| STAGE*sex | 3.18 | 4 | 0.80 | 1.18 | 3.17E-01 | 0.006 |
| STAGE*age_group | 38.72 | 12 | 3.23 | 4.80 | 1.19E-07 | 0.064 |
| STAGE*sex*age_group | 10.06 | 12 | 0.84 | 1.25 | 2.46E-01 | 0.018 |
| Error | 562.12 | 836 | 0.67 | | | |
| {4}REGION | 0.81 | 4 | 0.20 | 0.76 | 5.49E-01 | 0.004 |
| REGION*sex | 2.43 | 4 | 0.61 | 2.29 | 5.81E-02 | 0.011 |
| REGION*age_group | 13.69 | 12 | 1.14 | 4.30 | 1.23E-06 | 0.058 |
| REGION*sex*age_group | 5.45 | 12 | 0.45 | 1.71 | 5.99E-02 | 0.024 |
| Error | 221.99 | 836 | 0.27 | | | |
| {5}LEFT-RIG | 0.09 | 1 | 0.09 | 0.23 | 6.32E-01 | 0.001 |
| LEFT-RIG*sex | 0.92 | 1 | 0.92 | 2.44 | 1.19E-01 | 0.012 |
| LEFT-RIG*age_group | 1.89 | 3 | 0.63 | 1.68 | 1.72E-01 | 0.024 |
| LEFT-RIG*sex*age_group | 0.30 | 3 | 0.10 | 0.27 | 8.49E-01 | 0.004 |
| Error | 78.34 | 209 | 0.37 | | | |
| STAGE*REGION | 17.31 | 16 | 1.08 | 9.18 | 0.00E+00 | 0.042 |
| STAGE*REGION*sex | 3.96 | 16 | 0.25 | 2.10 | 6.27E-03 | 0.010 |
| STAGE*REGION*age_group | 14.03 | 48 | 0.29 | 2.48 | 8.57E-08 | 0.034 |
| STAGE*REGION*sex*age_group | 6.93 | 48 | 0.14 | 1.22 | 1.39E-01 | 0.017 |
| Error | 394.21 | 3344 | 0.12 | | | |
| STAGE*LEFT-RIG | 0.06 | 4 | 0.01 | 0.17 | 9.53E-01 | 0.001 |
| STAGE*LEFT-RIG*sex | 0.09 | 4 | 0.02 | 0.27 | 8.97E-01 | 0.001 |
| STAGE*LEFT-RIG*age_group | 1.39 | 12 | 0.12 | 1.38 | 1.67E-01 | 0.019 |
| 3*5*1*2 | 0.36 | 12 | 0.03 | 0.36 | 9.77E-01 | 0.005 |
| Error | 69.74 | 836 | 0.08 | | | |
| REGION*LEFT-RIG | 0.78 | 4 | 0.19 | 2.36 | 5.18E-02 | 0.011 |
| REGION*LEFT-RIG*sex | 0.26 | 4 | 0.07 | 0.79 | 5.30E-01 | 0.004 |
| REGION*LEFT-RIG*age_group | 1.29 | 12 | 0.11 | 1.30 | 2.10E-01 | 0.018 |
| 4*5*1*2 | 1.08 | 12 | 0.09 | 1.09 | 3.65E-01 | 0.015 |
| Error | 68.88 | 836 | 0.08 | | | |
| STAGE*REGION*LEFT-RIG | 0.59 | 16 | 0.04 | 0.77 | 7.26E-01 | 0.004 |
| STAGE*REGION*LEFT-RIG*sex | 0.76 | 16 | 0.05 | 0.99 | 4.64E-01 | 0.005 |
| 3*4*5*2 | 3.60 | 48 | 0.07 | 1.56 | 8.59E-03 | 0.022 |

### 1.6.3 Peak center frequency

| Effect | Repeated Measures Analysis of Variance with Effect Sizes Sigma-restricted parameterization Effective hypothesis decomposition SS | df | MS | F | p | $\eta_p^2$ |
|---|---|---|---|---|---|---|
| Intercept | 969134.83 | 1 | 969134.83 | 3540.34 | 0.00E+00 | 0.945 |
| {1}sex | 11.71 | 1 | 11.71 | 0.04 | 8.36E-01 | 0.000 |
| {2}age_group | 14124.78 | 3 | 4708.26 | 17.20 | 5.25E-10 | 0.200 |
| sex*age_group | 116.45 | 3 | 38.82 | 0.14 | 9.35E-01 | 0.002 |
| Error | 56390.64 | 206 | 273.74 | | | |
| {3}STAGE | 4795.75 | 4 | 1198.94 | 15.58 | 2.68E-12 | 0.070 |
| STAGE*sex | 284.57 | 4 | 71.14 | 0.92 | 4.49E-01 | 0.004 |
| STAGE*age_group | 4351.01 | 12 | 362.58 | 4.71 | 1.79E-07 | 0.064 |
| STAGE*sex*age_group | 903.02 | 12 | 75.25 | 0.98 | 4.68E-01 | 0.014 |
| Error | 63391.51 | 824 | 76.93 | | | |
| {4}REGION | 5802.22 | 4 | 1450.55 | 58.14 | 0.00E+00 | 0.220 |
| REGION*sex | 87.81 | 4 | 21.95 | 0.88 | 4.75E-01 | 0.004 |
| REGION*age_group | 787.97 | 12 | 65.66 | 2.63 | 1.85E-03 | 0.037 |
| REGION*sex*age_group | 459.39 | 12 | 38.28 | 1.53 | 1.06E-01 | 0.022 |
| Error | 20559.03 | 824 | 24.95 | | | |
| {5}LEFT-RIG | 8.56 | 1 | 8.56 | 1.09 | 2.98E-01 | 0.005 |
| LEFT-RIG*sex | 11.49 | 1 | 11.49 | 1.46 | 2.28E-01 | 0.007 |
| LEFT-RIG*age_group | 23.17 | 3 | 7.72 | 0.98 | 4.03E-01 | 0.014 |
| LEFT-RIG*sex*age_group | 28.16 | 3 | 9.39 | 1.19 | 3.14E-01 | 0.017 |
| Error | 1622.61 | 206 | 7.88 | | | |
| STAGE*REGION | 5388.42 | 16 | 336.78 | 23.31 | 0.00E+00 | 0.102 |
| STAGE*REGION*sex | 349.72 | 16 | 21.86 | 1.51 | 8.58E-02 | 0.007 |
| STAGE*REGION*age_group | 1936.19 | 48 | 40.34 | 2.79 | 8.17E-10 | 0.039 |
| STAGE*REGION*sex*age_group | 780.69 | 48 | 16.26 | 1.13 | 2.57E-01 | 0.016 |
| Error | 47611.82 | 3296 | 14.45 | | | |
| STAGE*LEFT-RIG | 40.02 | 4 | 10.00 | 1.49 | 2.03E-01 | 0.007 |
| STAGE*LEFT-RIG*sex | 87.58 | 4 | 21.89 | 3.27 | 1.14E-02 | 0.016 |
| STAGE*LEFT-RIG*age_group | 63.51 | 12 | 5.29 | 0.79 | 6.62E-01 | 0.011 |
| 3*5*1*2 | 72.32 | 12 | 6.03 | 0.90 | 5.48E-01 | 0.013 |
| Error | 5523.86 | 824 | 6.70 | | | |
| REGION*LEFT-RIG | 58.20 | 4 | 14.55 | 2.48 | 4.25E-02 | 0.012 |
| REGION*LEFT-RIG*sex | 19.45 | 4 | 4.86 | 0.83 | 5.06E-01 | 0.004 |
| REGION*LEFT-RIG*age_group | 100.43 | 12 | 8.37 | 1.43 | 1.47E-01 | 0.020 |
| 4*5*1*2 | 60.64 | 12 | 5.05 | 0.86 | 5.86E-01 | 0.012 |
| Error | 4829.67 | 824 | 5.86 | | | |
| STAGE*REGION*LEFT-RIG | 104.52 | 16 | 6.53 | 1.33 | 1.72E-01 | 0.006 |
| STAGE*REGION*LEFT-RIG*sex | 72.92 | 16 | 4.56 | 0.92 | 5.40E-01 | 0.004 |
| 3*4*5*2 | 303.60 | 48 | 6.32 | 1.28 | 9.19E-02 | 0.018 |
| 3*4*5*1*2 | 206.51 | 48 | 4.30 | 0.87 | 7.19E-01 | 0.013 |
| Error | 16244.15 | 3296 | 4.93 | | | |

### 1.6.4 Peak power

| Effect | Repeated Measures Analysis of Variance with Effect Size<br>Sigma-restricted parameterization<br>Effective hypothesis decomposition | | | | | |
|---|---|---|---|---|---|---|
| | SS | df | MS | F | p | $\eta_p^2$ |
| Intercept | 2909.38 | 1 | 2909.38 | 3215.24 | 0.00E+00 | 0.940 |
| {1}sex | 0.09 | 1 | 0.09 | 0.09 | 7.58E-01 | 0.000 |
| {2}age_group | 12.00 | 3 | 4.00 | 4.42 | 4.89E-03 | 0.060 |
| sex*age_group | 0.77 | 3 | 0.26 | 0.28 | 8.36E-01 | 0.004 |
| Error | 186.40 | 206 | 0.90 | | | |
| {3}STAGE | 112.52 | 4 | 28.13 | 88.77 | 0.00E+00 | 0.301 |
| STAGE*sex | 0.64 | 4 | 0.16 | 0.50 | 7.34E-01 | 0.002 |
| STAGE*age_group | 18.58 | 12 | 1.55 | 4.88 | 8.01E-08 | 0.066 |
| STAGE*sex*age_group | 5.52 | 12 | 0.46 | 1.45 | 1.37E-01 | 0.021 |
| Error | 261.12 | 824 | 0.32 | | | |
| {4}REGION | 19.08 | 4 | 4.77 | 97.65 | 0.00E+00 | 0.322 |
| REGION*sex | 0.32 | 4 | 0.08 | 1.64 | 1.61E-01 | 0.008 |
| REGION*age_group | 2.41 | 12 | 0.20 | 4.11 | 2.93E-06 | 0.056 |
| REGION*sex*age_group | 0.26 | 12 | 0.02 | 0.44 | 9.47E-01 | 0.006 |
| Error | 40.25 | 824 | 0.05 | | | |
| {5}LEFT-RIG | 0.00 | 1 | 0.00 | 0.01 | 9.40E-01 | 0.000 |
| LEFT-RIG*sex | 0.01 | 1 | 0.01 | 0.68 | 4.09E-01 | 0.003 |
| LEFT-RIG*age_group | 0.05 | 3 | 0.02 | 1.37 | 2.52E-01 | 0.020 |
| LEFT-RIG*sex*age_group | 0.01 | 3 | 0.00 | 0.17 | 9.17E-01 | 0.002 |
| Error | 2.63 | 206 | 0.01 | | | |
| STAGE*REGION | 14.20 | 16 | 0.89 | 47.27 | 0.00E+00 | 0.187 |
| STAGE*REGION*sex | 0.10 | 16 | 0.01 | 0.33 | 9.95E-01 | 0.002 |
| STAGE*REGION*age_group | 5.95 | 48 | 0.12 | 6.60 | 0.00E+00 | 0.088 |
| STAGE*REGION*sex*age_group | 1.01 | 48 | 0.02 | 1.12 | 2.58E-01 | 0.016 |
| Error | 61.90 | 3296 | 0.02 | | | |
| STAGE*LEFT-RIG | 0.10 | 4 | 0.03 | 4.89 | 6.67E-04 | 0.023 |
| STAGE*LEFT-RIG*sex | 0.02 | 4 | 0.01 | 1.04 | 3.88E-01 | 0.005 |
| STAGE*LEFT-RIG*age_group | 0.17 | 12 | 0.01 | 2.71 | 1.31E-03 | 0.038 |
| 3*5*1*2 | 0.03 | 12 | 0.00 | 0.49 | 9.21E-01 | 0.007 |
| Error | 4.33 | 824 | 0.01 | | | |
| REGION*LEFT-RIG | 0.04 | 4 | 0.01 | 1.18 | 3.18E-01 | 0.006 |
| REGION*LEFT-RIG*sex | 0.03 | 4 | 0.01 | 1.03 | 3.92E-01 | 0.005 |
| REGION*LEFT-RIG*age_group | 0.13 | 12 | 0.01 | 1.29 | 2.17E-01 | 0.018 |
| 4*5*1*2 | 0.11 | 12 | 0.01 | 1.08 | 3.72E-01 | 0.016 |
| Error | 6.92 | 824 | 0.01 | | | |
| STAGE*REGION*LEFT-RIG | 0.13 | 16 | 0.01 | 2.46 | 9.93E-04 | 0.012 |
| STAGE*REGION*LEFT-RIG*sex | 0.07 | 16 | 0.00 | 1.29 | 1.91E-01 | 0.006 |
| 3*4*5*2 | 0.29 | 48 | 0.01 | 1.85 | 3.70E-04 | 0.026 |
| 3*4*5*1*2 | 0.22 | 48 | 0.00 | 1.38 | 4.48E-02 | 0.020 |
| Error | 10.82 | 3296 | 0.00 | | | |

### 1.6.5 Adjusted spectral slope

| Effect | Repeated Measures Analysis of Variance with Effect Sizes Sigma-restricted parameterization Effective hypothesis decomposition | | | | | |
|---|---|---|---|---|---|---|
| | SS | df | MS | F | p | $\eta_p^2$ |
| Intercept | 1688.622 | 1 | 1688.622 | 492.449 | 0.00E+00 | 0.702045 |
| {1}sex | 0.664 | 1 | 0.664 | 0.194 | 6.60E-01 | 0.000925 |
| {2}age_group | 36.531 | 3 | 12.177 | 3.551 | 1.53E-02 | 0.048501 |
| Error | 716.667 | 209 | 3.429 | | | |
| {3}STAGE | 552.469 | 3 | 184.156 | 1198.557 | 0.00E+00 | 0.851516 |
| STAGE*sex | 0.240 | 3 | 0.080 | 0.520 | 6.68E-01 | 0.002484 |
| STAGE*age_group | 16.050 | 9 | 1.783 | 11.607 | 1.11E-16 | 0.142812 |
| Error | 96.338 | 627 | 0.154 | | | |
| {4}REGION | 8.766 | 4 | 2.191 | 43.498 | 0.00E+00 | 0.172270 |
| REGION*sex | 0.321 | 4 | 0.080 | 1.592 | 1.74E-01 | 0.007559 |
| REGION*age_group | 1.485 | 12 | 0.124 | 2.456 | 3.76E-03 | 0.034051 |
| Error | 42.117 | 836 | 0.050 | | | |
| {5}LEFT-RIG | 0.066 | 1 | 0.066 | 1.086 | 2.98E-01 | 0.005171 |
| LEFT-RIG*sex | 0.241 | 1 | 0.241 | 3.936 | 4.86E-02 | 0.018485 |
| LEFT-RIG*age_group | 0.411 | 3 | 0.137 | 2.241 | 8.45E-02 | 0.031172 |
| Error | 12.782 | 209 | 0.061 | | | |
| STAGE*REGION | 3.834 | 12 | 0.319 | 62.999 | 0.00E+00 | 0.231614 |
| STAGE*REGION*sex | 0.080 | 12 | 0.007 | 1.316 | 2.02E-01 | 0.006255 |
| STAGE*REGION*age_group | 1.201 | 36 | 0.033 | 6.577 | 0.00E+00 | 0.086263 |
| Error | 12.718 | 2508 | 0.005 | | | |
| STAGE*LEFT-RIG | 0.027 | 3 | 0.009 | 1.295 | 2.75E-01 | 0.006156 |
| STAGE*LEFT-RIG*sex | 0.005 | 3 | 0.002 | 0.260 | 8.54E-01 | 0.001241 |
| STAGE*LEFT-RIG*age_group | 0.055 | 9 | 0.006 | 0.869 | 5.52E-01 | 0.012326 |
| Error | 4.402 | 627 | 0.007 | | | |
| REGION*LEFT-RIG | 0.098 | 4 | 0.024 | 1.729 | 1.42E-01 | 0.008204 |
| REGION*LEFT-RIG*sex | 0.013 | 4 | 0.003 | 0.236 | 9.18E-01 | 0.001128 |
| REGION*LEFT-RIG*age_group | 0.129 | 12 | 0.011 | 0.762 | 6.90E-01 | 0.010826 |
| Error | 11.830 | 836 | 0.014 | | | |
| STAGE*REGION*LEFT-RIG | 0.010 | 12 | 0.001 | 0.621 | 8.26E-01 | 0.002964 |
| STAGE*REGION*LEFT-RIG*sex | 0.015 | 12 | 0.001 | 0.967 | 4.79E-01 | 0.004603 |
| 3*4*5*2 | 0.046 | 36 | 0.001 | 0.997 | 4.75E-01 | 0.014104 |
| Error | 3.220 | 2508 | 0.001 | | | |

## REFERENCES


Vallat, R. and Walker, M. P. (2021). An open-source, high-performance tool for automated sleep staging. *eLife* 10. doi:10.7554/elife.70092

Wen, H. and Liu, Z. (2015). Separating fractal and oscillatory components in the power spectrum of neurophysiological signal. *Brain Topography* 29, 13–26. doi:10.1007/s10548-015-0448-0
